# Gastric cancer vaccines synthesized using a TLR7 agonist and their synergistic antitumor effects with 5-fluorouracil

**DOI:** 10.1186/s12967-018-1501-z

**Published:** 2018-05-08

**Authors:** Xiaodong Wang, Yu Liu, Yuwen Diao, Ningning Gao, Yanyan Wan, Jingjing Zhong, Huali Zheng, Zhulin Wang, Guangyi Jin

**Affiliations:** 1 School of Pharmaceutical Sciences, Shenzhen University Health Science Center, Shenzhen, 518060 Guangdong China; 2 Cancer Research Center, Shenzhen University Health Science Center, Shenzhen, 518060 Guangdong China; 3 The 3rd Affiliated Hospital of Shenzhen University, Shenzhen, 518001 Guangdong China; 4 Department of Biology and School of Medicine, Southern University of Science and Technology, Shenzhen, 518055 Guangdong China

**Keywords:** Gastric cancer, Vaccine, TLR7, MG7-Ag, 5-Fluorouracil

## Abstract

**Background:**

Vaccines play increasingly important roles in cancer treatment due to their advantages of effective targeting and few side effects. Our laboratory has attempted to construct vaccines by conjugating TLR7 agonists with tumor-associated antigens. Furthermore, immunochemotherapy has recently become an appealing approach to cancer therapy. 5-fluorouracil (5-FU), a commonly used chemotherapeutic agent, can reportedly potently and selectively kill tumor-associated MDSCs in vivo.

**Methods:**

Gastric cancer vaccines were synthesized by the covalent attachment of our TLR7 agonist with the gastric cancer antigen MG7-Ag tetra-epitope, leading to T7 − ML (linear tetra-epitope) and T7 − MB (branched tetra-epitope). Cytokines induced by the vaccines in vitro were assessed by ELISA. A tumor challenge model was created by treating BALB/c mice on either a prophylactic or therapeutic vaccination schedule. 5-FU was simultaneously applied to mice in the combination treatment group. CTL and ADCC activities were determined by the LDH method, while CD3^+^/CD8^+^, CD3^+^/CD4^+^ T cells and MDSCs were evaluated by flow cytometry.

**Results:**

In vitro, rapid TNF-α and IL-12 inductions occurred in BMDCs treated with the vaccines. In vivo, among all the vaccines tested, T7 − MB most effectively reduced EAC tumor burdens and induced CTLs, antibodies and ADCC activity in BALB/c mice. Immunization with T7 − MB in combination with 5-FU chemotherapy reduced tumor sizes and extended long-term survival rates, mainly by improving T cell responses, including CTLs, CD3^+^/CD8^+^ and CD3^+^/CD4^+^ T cells. 5-FU also enhanced the T7 − MB efficiency by reversing immunosuppressive factors, i.e., MDSCs, which could not be validly inhibited by the vaccines alone. In addition, T7 − MB repressed tumor growth and immune tolerance when the therapeutic schedule was used, although the effects were weaker than those achieved with either T7 − MB alone or in combination with 5-FU on the prophylactic schedule.

**Conclusions:**

A novel effective gastric cancer vaccine was constructed, and the importance of branched multiple antigen peptides and chemical conjugation to vaccine design were confirmed. The synergistic effects and mechanisms of T7 − MB and 5-FU were also established, observing mainly T cell activation and MDSC inhibition.

**Electronic supplementary material:**

The online version of this article (10.1186/s12967-018-1501-z) contains supplementary material, which is available to authorized users.

## Background

Toll-like receptors (TLRs), expressed in a variety of immune cells, are recognized for their ability to initiate immune responses upon exposure to specific pathogen-associated molecular patterns (PAMPs), including bacterial lipopolysaccharide (LPS) and flagellin [1]. Recently, TLRs have also been closely related to immune activation against diverse cancers, as at least three types of TLR agonists have been approved by the FDA for use in cancer treatment: bacillus Calmette–Guérin, monophosphoryl lipid A (MPL) and imiquimod [2]. Imiquimod, a typical small-molecule TLR7 agonist with a non-nucleoside imidazoquinoline structure, was approved for the treatment of patients with superficial basal cell carcinoma in 2004 [3] and has also been applied as an adjuvant in investigations on antitumor vaccines. A phase II trial demonstrated that imiquimod/TA-CIN (a recombinant HPV fusion protein vaccine) engendered T cell infiltration and lesion regression [4]. Imiquimod also exerts therapeutic effects on chronic myeloid leukemia (CML) patients when administered together with a granulocyte macrophage-colony stimulating factor (GM-CSF) producing-vaccine derived from a CML cell line [5]. Activation of TLR7 signaling in immune cells depends mostly on myeloid differentiation factor 88 (MyD88) and downstream transcription factors, such as members of the interferon (IFN)-regulatory factor (IRF) family and nuclear factor-κB (NF-κB), resulting in the release of stimulatory molecules and immune system stimulation [6]. In addition to imiquimod, UC-1V150 is another well-studied TLR7 agonist that increases the levels of proinflammatory cytokines by conjugation to phospholipids [7]. We have constructed a series of vaccines by covalently attaching a small-molecule TLR7 agonist to a gastric cancer antigen, leading to immunogenicity stimulation and tumor inhibition when introduced to animal models [8]. Our laboratory recently synthesized a novel TLR7 agonist (T7) that enhanced the effectiveness of chemotherapeutic agents in a murine model of T cell lymphoma [9].

Tumor-associated antigens (TAAs) are specifically expressed in tumor cells and can be recognized by the immune system and applied as immunotherapy targets against cancer [10]. Some important tumor antigens have been detected to evaluate the diagnosis and prognosis of gastric cancer for decades, such as CEA (carcino-embryonic antigen), CA19-9 (carbohydrate antigen 19-9) and CA125 (carbohydrate antigen 125) [11]. Monoclonal gastric cancer 7 antigen (MG7-Ag) is a TAA in gastric cancer with a much higher specificity and selectivity than those of previously discovered antigens. MG7-Ag is substantially important in the progression of a tumor, whose expression in gastric mucosa is highly likely to be linked with atypical hyperplasia and malignant changes [12, 13].

In this study, we constructed novel gastric cancer vaccines using collaborative applications of our TLR7 agonist (T7) and the MG7-Ag tetra-epitope. Herein, using multiple antigen peptides (MAPs) of the MG7-Ag epitope, with an especially branched architecture, substantially improved our previous vaccine designs. The efficiencies of vaccines on eliciting humoral and cellular immune responses were demonstrated by cytokine detection, antibody titer determination, antibody-dependent cell-mediated cytotoxicity (ADCC) and cytotoxic T lymphocyte (CTL) activity and flow cytometry. A tumor challenge assay was also carried out to confirm that T7 − MB (conjugation of T7 and the MG7-Ag branched tetra-epitope) was the most potent agent in reversing tumor tolerance. Next, the synergistic effects of T7 − MB and 5-fluorouracil (5-FU, a commonly used chemotherapeutic agent), administered on both prophylactic and therapeutic vaccination schedules, on tumor inhibition were displayed by variations in tumor volumes, tumor weights and long-term survivals.

## Methods

### Chemical synthesis

T7 was synthesized as described before [9]. For the syntheses of peptides, 1.0 mmol/g 2-chlorotrityl chloride resin (GL BIO Company, Shanghai, China) was loaded as the solid support, and the following N-protected Fmoc amino acids were used as the functionalized amino acids: Fmoc-Lys(Boc)-OH, Fmoc-Thr(tBu)-OH, Fmoc-His(Trt)-OH, Fmoc-Val-OH, Fmoc-Pro-OH (GL BIO Company) and T7. The coupling reagent was 2-(1,H-benzotriazol-1-yl)-1,1,3,3-tetramethyluronium hexafluorophosphate. Fmoc-Lys(Boc)-OH was first coupled to the resin, while successive amino acids were applied at 1 or 4 equal molar quantities for synthesis of the MG7-Ag linear tetra-epitope (ML) or the MG7-Ag branched tetra-epitope (MB). Fmoc deprotection was performed in a solution containing piperidine and dimethylformamide in a ratio of 2/8 (v/v). Disaggregation of the peptides from the resin was implemented in a mixture of trifluoroacetic acid, phenol, water and triisopropylsilane in a ratio of 88/5/5/2 (v/v) for 3.5 h. The harvested peptides were precipitated by cold diethyl ether, dissolved by 0.1% trifluoroacetic acid in water/acetonitrile and lyophilized. Finally, electrospray mass spectrometry was completed to identify the structures of the peptides, and analytical high-performance liquid chromatography (C18 column, 5 µm, 300 Å, 10.0 × 200 mm) was used to further confirm a purity of at least 95% (UV detection at 214 and 254 nm).

### Cytokine assays

Mouse bone marrow dendritic cells (BMDCs) were generated from the femurs and tibias of BALB/c mice as described previously [8]. BMDCs were cultured in X-vivo 15 medium (Lonza, Walkersville, WV, USA) containing 10 ng/mL GM-CSF and 10 ng/mL IL-4 at 37 °C for 6 days and then seeded in 96-well plates at a density of 5 × 10^4^ cells per well. Vaccines were added at a final concentration of 5 µM or 10 µM and incubated for 24 h. The cytokines TNF-α and IL-12 in the culture supernatants were quantified using Mouse TNF-α and IL-12 p70 ELISA Ready-SET-Go reagent sets (eBioscience, San Diego, CA, USA) according to the manufacturer’s specifications. Briefly, an ELISA plate was first coated at 4 °C overnight with a capture antibody and then successively loaded with a blocking solution, the culture supernatant and a detection antibody for 1 h at room temperature. Finally, the substrate and stop solution were filled to obtain the optical density, which was recorded with a spectrophotometer (BioTek, Winooski, VT, USA) at 450 nm.

### Tumor challenge model

The animal protocol, approved by the Laboratory Animal Ethics Committee of Shenzhen University, was proposed to attenuate animal discomfort. Female 4-week-old (for prophylactic vaccination) and 6-week-old BALB/c (for therapeutic vaccination) mice were purchased from the Medical Laboratory Animal Center (Guangzhou, Guangdong, China). All mice were housed in specific pathogen-free conditions on a 12 h light/dark cycle, provided water and food ad libitum, and acclimated for 1 week before the formal experiments. Each BALB/c mouse was subcutaneously challenged on the right-hand side of its back with 1 × 10^7^ ehrlich ascites carcinoma (EAC) cells, in which MG7-Ag expression was validated by western blot using an MG7-Ag antibody (a gift from Dr. Fan Daiming and Dr. Nie Yongzhan).

### Prophylactic vaccination schedule

**Fig. 1:**
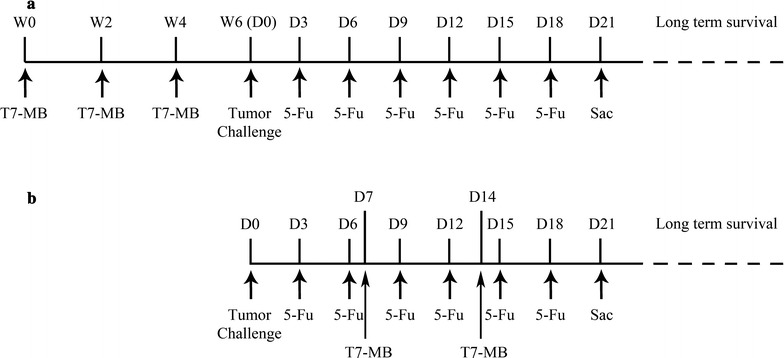
Schematic diagram illustrating the prophylactic vaccination (**a**) and therapeutic vaccination (**b**) schedules. Mice were injected with 100 µg of T7 − MB (s.c.) and 30 mg/kg 5-FU (i.p.) (W: week; D: day)

BALB/c mice were immunized with the vaccines, and PBS served as the control. Each mouse was subcutaneously administered 100 µg of T7 − MB or equal molar quantities of the other vaccines every 2 weeks three times and challenged with EAC cells on day 0. Furthermore, 30 mg/kg 5-FU was applied intraperitoneally every 3 days after the tumor challenge until day 21 (Fig. 1a). Tumor dimensions were measured with Vernier calipers every 3 days, and tumor volumes were calculated with the formula (A × B^2^)/2, where A and B represented the larger and smaller dimensions of the tumor, respectively. All mice were sacrificed on day 21, and the tumors were removed and weighed. The tumor weights in each group were expressed as the percentages relative to the PBS control. Long-term survival was also evaluated until the mice died naturally.

### Therapeutic vaccination schedule

BALB/c mice were first subcutaneously challenged with EAC cells on day 0. Next, 100 µg of T7 − MB or equal molar quantities of the other vaccines were administered on days 7 and 14, while 30 mg/kg 5-FU was administered every 3 days until day 21 (Fig. 1b). Tumor dimensions, tumor weights and long-term survival rates were determined as described above.

### Determinations of CTLs

Lymphocytes (effectors) were derived from the spleens of mice using Mouse Lymphocyte Separation Medium (Dakewe, Beijing, China) at the time of sacrifice. EAC tumor cells (targets) were co-cultured with lymphocytes at an effector-to-target cell ratio of 50:1 for 4 h. Cytotoxicity was determined by the lactate dehydrogenase (LDH) method using the non-radioactive cytotoxicity assay (Promega, Madison, WI, USA) according to the manufacturer’s specifications. Shortly thereafter, an ELISA plate was loaded with the culture supernatant, and substrate solution was added at room temperature for 30 min. Finally, a stop solution was filled to obtain the optical density, which was recorded with a spectrophotometer (BioTek) at 490 nm.

### Determinations of antibody titers

Blood samples were collected from the mice after each immunization on the prophylactic vaccination schedule to obtain serum samples by centrifugation at 3000 g for 15 min. Antibody titers were quantified by the ELISA method using an alkaline phosphate-conjugated detection antibody for total IgG (Millipore, Billerica, MA, USA). Briefly, an ELISA plate was first coated at 4 °C overnight with BSA-MG1 (sequence is BSA-KPHVHTK) and later successively loaded with a blocking solution for 2 h at room temperature, the serum samples (1:50 diluted) and a detection antibody for 1 h. Finally, the p-NPP substrate (Millipore) and stop solution were filled to obtain the optical density, which was recorded with a spectrophotometer (BioTek) at 405 nm.

### Determinations of ADCC activity

Serum samples (1:25 diluted) were isolated from the mice at the time of sacrifice and then incubated with EAC tumor cells (targets) for 30 min at 37 °C. Natural killer (NK) cells (effectors), separated from a normal BALB/c mouse using a Mouse NK Cell Separation Kit (Hao Yang, Tianjin, China), were co-cultured with antibody-labeled EAC cells at an effector-to-target cell ratio of 50:1 for 4 h. Cytotoxicity was determined by the LDH method using the non-radioactive cytotoxicity assay (Promega) according to the specifications described above.

### Flow cytometry

At the time of sacrifice, the mouse spleens were homogenized by repeated pipetting and filtered through a 70-μm nylon filter. Single-cell splenocyte suspensions were washed three times with FACS, stained with the appropriate antibodies at a final concentration of 1 μg/ml at 4 °C overnight, and analyzed by FACSCalibur flow cytometry (BD Biosciences, San Jose, CA, USA). The following antibodies, purchased from eBioscience, were used for flow cytometry: CD3-Alexa Fluor 488, CD4-PerCP Cy5.5, CD8a-PE, CD11b-Alexa Fluor 647 and Gr-1-PerCP Cy5.5.

### Statistical analysis

Data are expressed as the means ± SEs for the indicated numbers of independently performed experiments. One-way ANOVA was used for the determination of statistical significance, and differences were considered statistically significant at *P *< 0.05.

## Results

### Chemical synthesis and in vitro cytokine release of vaccines

**Fig. 2:**
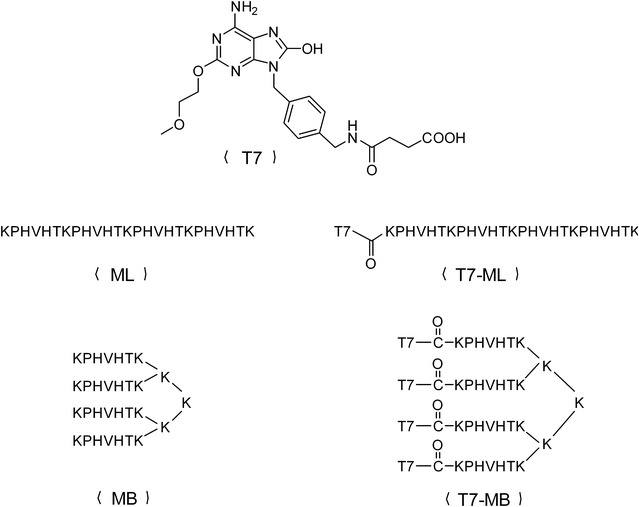
Chemical structures of the synthetic vaccines

**Fig. 3:**
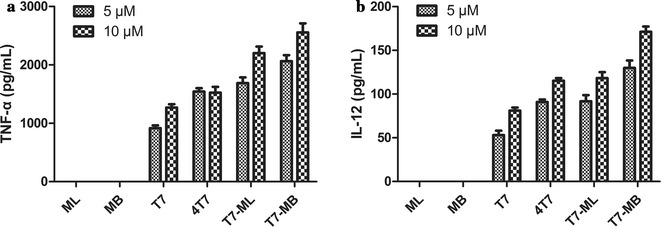
Vaccines induced the cytokine release of mouse bone marrow dendritic cells (BMDCs) in vitro. BMDCs of BALB/c mice were incubated with the vaccines for 24 h, and mouse TNF-α (**a**) and IL-12 (**b**) levels were quantified by ELISA. 4T7 represents the 4-fold concentration of T7, which was compared with T7 − MB since 4 mol of T7 were conjugated to 1 mol of MB in T7 − MB. The data represent the mean ± SE (*n *= 3)

A novel TLR7 agonist (T7) (molecular weight: 444) was synthesized by our group as previously described [9] and used in the preparation of the other vaccines in this study (Fig. 2). Four peptides were obtained by the solid phase method using an Fmoc strategy: ML (molecular weight: 2945), MB (molecular weight: 3714), conjugation of T7 and ML (T7 − ML) (molecular weight: 3372), and conjugation of T7 and MB (T7 − MB) (molecular weight: 5420). The conjugations were constructed by covalent attachment of the carboxyl group in T7 and the amino group in the nitrogen terminus of peptides. Mass spectrometry and high-performance liquid chromatography were applied to confirm the structures and purities of the above compounds (Additional files 1, 2, 3, 4). Moreover, T7 and the MG7-Ag epitope were also assembled by commixing T7 and ML at a 1:1 ratio (T7 + ML) and T7 and MB at a 4:1 ratio (T7 + MB). Indicated concentrations of the vaccines were incubated with mouse BMDCs to determine their cytokine production abilities (Fig. 3). When BMDCs were exposed to ML or MB alone, no initiation of two cytokines (TNF-α and IL-12) was detected. T7 dose-dependently increased TNF-α and IL-12 from 5 to 40 µM (5–10 µM 4T7 is equal to 20–40 µM T7). Compared with T7 and 4T7, distinctly higher levels of cytokines were recognized in the T7 − ML and T7 − MB groups, i.e., the T7 and MB conjugation was much more potent than T7 or MB alone. Furthermore, the conjugated branched epitope had a stronger stimulating ability on BMDCs than the linear epitope, as the TNF-α and IL-12 levels were more efficiently enhanced by the T7 − MB group than by the T7 − ML group.

### Tumor inhibition and immunogenicity stimulation of the vaccines

**Fig. 4:**
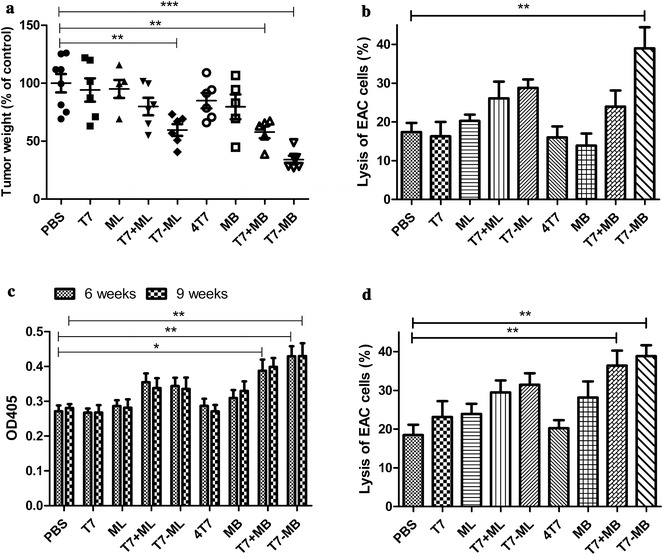
Effects of the vaccines on tumor inhibition and immunogenicity stimulation in BALB/c mice (n ≥ 5/group). **a** EAC tumor burdens were reduced by the vaccines, where the PBS control (100%) was 1.06 ± 0.08 g. **b** Cytotoxic T cell responses were induced by the vaccines, as indicated by the lysis of EAC cells using the LDH method. **c** Serum antibodies against MG7-Ag induced by the vaccines after immunization (6 weeks) and after sacrifice (9 weeks), as determined by ELISA. **d** Antibody-dependent cell-mediated cytotoxicity (ADCC) effects induced by the vaccines, as indicated by the lysis of EAC cells using the LDH method. The data represent the mean ± SE; *P < 0.05, **P < 0.01 and ***P < 0.001 compared to the PBS control group

A tumor challenge model was established in vivo by the subcutaneous inoculation of EAC cells into BALB/c mice to explore the prophylactic abilities of the vaccines. Herein, EAC cells represented a type of mouse ascites carcinoma that expresses MG7-Ag at high levels, which was affirmed by western blot (Additional file 5). Four equal molar quantities of T7 (4T7) contrasted the branched epitope groups (T7 + MB and T7 − MB). Compared with the PBS control, immunization with T7, ML or MB alone every 2 weeks three times had no apparent effect on tumor development. Reductions in tumor weights were clearly observed when T7 was used in combination with the MG7-Ag tetra-epitope (ML or MB) (Fig. 4a). In addition, comparing four vaccines (T7 + ML, T7 − ML, T7 + MB and T7 − MB) showed that the branched epitope better improved the antitumor properties than the linear epitope, while T7 and the epitope in chemical conjugation was more efficient than the simple commixture. Next, CTL activity was measured to determine cellular immunity activations in vaccinated BALB/c mice. As shown in Fig. 4b, T7 and the MG7-Ag tetra-epitope in combination had a significantly higher lytic activity than the PBS control, whereas the lytic activities of T7, ML alone and MB alone did not differ from that of the control. Similar to the tumor inhibition results, T7 − MB, constructed with the branched epitope and chemical conjugation, had the highest cytotoxicity among all the vaccines. For the impacts on humoral immunity, serum antibody titers of the vaccinations against MG7-Ag were first judged by the ELISA method. No distinct differences in antibodies were detected at 2 or 4 weeks after first immunization (data not shown). Six and nine weeks later, the MG7-Ag antibodies displayed marked increases in the T7 + ML, T7 − ML, T7 + MB and T7 − MB groups, and the vaccines constructed with MB elicited more potent responses than those constructed with ML. However, the differences between 6 and 9 weeks and between simple commixture and chemical conjugation were negligible (Fig. 4c). ADCC activity was also assessed by adding NK cells and serum samples to tumor cells. Antibodies derived from T7 − MB induced the most powerful cancer cell lysis, but the differences in ADCC activity between the simple commixture (T7 + MB) and chemical conjugation (T7 − MB) samples were not as significant as those between their CTL activities (Fig. 4d). No remarkable CTL or ADCC effects on the vaccines were found when mouse breast cancer 4T1 cells were used as target cells for the negative control (data not shown).

### Synergistic effects of 5-FU and T7 − MB on tumor inhibition using the prophylactic schedule

**Fig. 5:**
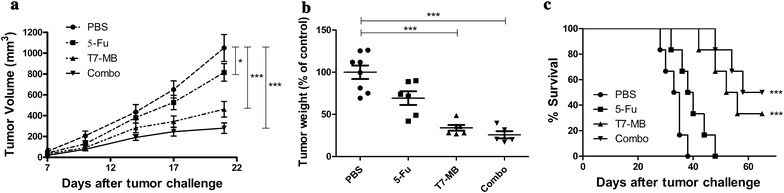
Additive effects of 5-FU and T7 − MB on tumor inhibition using the prophylactic schedule (n ≥ 5 mice/group). **a** Tumor growth curves displaying tumor volumes, where the dimensions were measured twice a week until day 21 (two-way ANOVA). **b** Tumor weights were determined at the time of sacrifice on day 21, where the PBS control (100%) was 1.06 ± 0.08 g. **c** Survival curves indicating that post-treatment effects were exerted on tumor-bearing mice for more than 70 days (log-rank test). The data represent the mean ± SE; *P < 0.05 and ***P < 0.001 compared to the PBS control group

Since T7 − MB was the most effective vaccine in this study, the synergistic effects of T7 − MB and 5-FU (a typical chemotherapeutic agent) on tumor challenge were further examined in vivo. Substantially more potent inhibitory effects on tumor sizes were observed upon the combination treatment of 5-FU and T7 − MB administered on the prophylactic schedule compared with those observed with control or single treatments. As shown in Fig. 5a, T7 − MB inhibited tumor volumes more efficiently than 5-FU in EAC tumor-challenged mice, and further shrinkage was increasingly clear upon the simultaneous administration of 5-FU and T7 − MB from days 1 through 21. Regarding tumor weights, 25.82% of the combination treatment group was statistically significantly different from the control group (100.00%), the 5-FU group (69.26%) and the T7 − MB group (34.02%) (Fig. 5b). Figure 5c shows that the first mouse death was reported in the control group on day 28. The last deaths in the control and 5-FU groups occurred on days 38 and 48, respectively. Compared with the T7 − MB group (33.33% mice surviving longer than 70 days), the combination treatment group (50.00%) exhibited substantially increased long-term survival rates during a 70-day observation period.

**Fig. 6:**
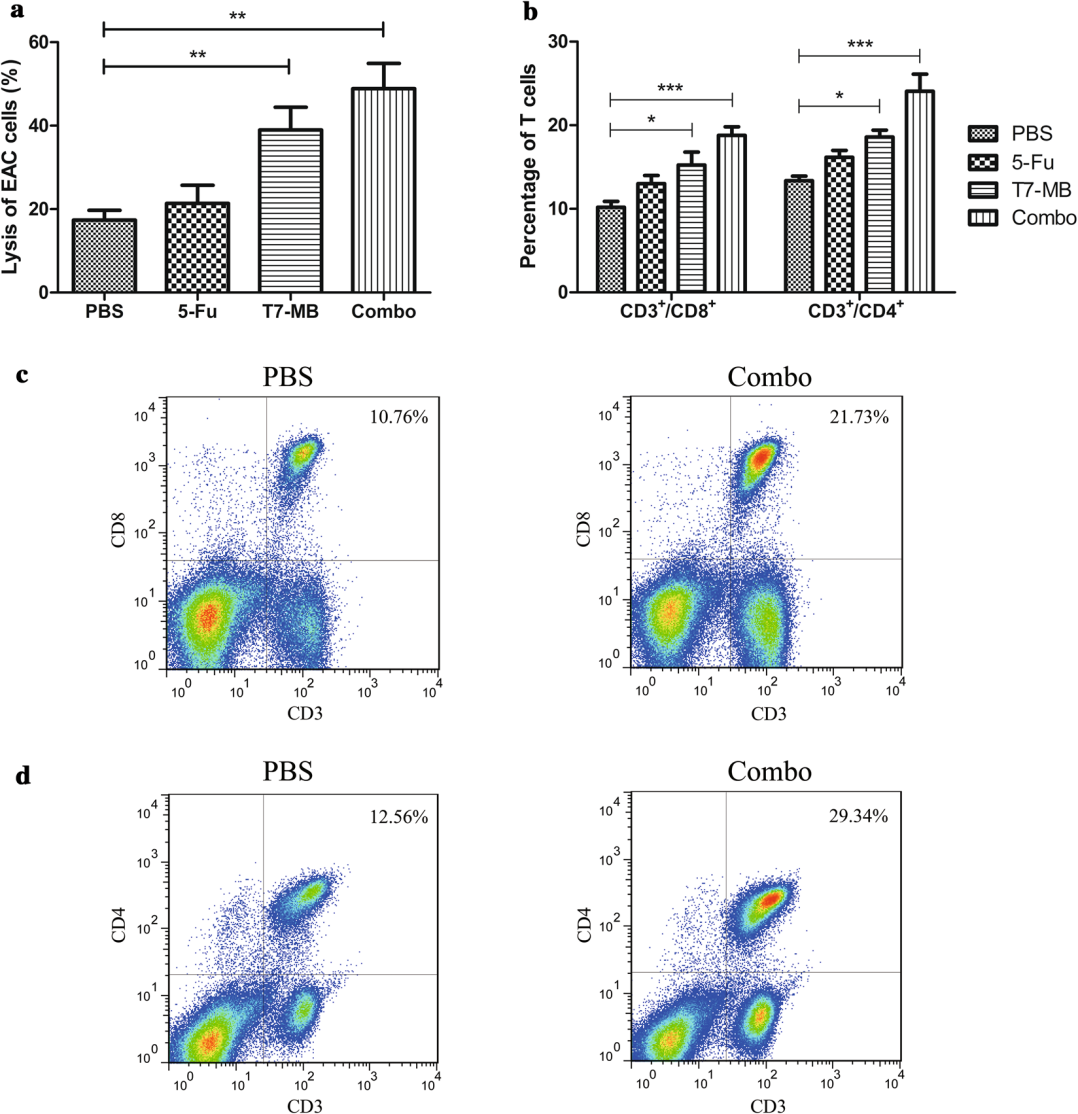
Additive effects of 5-FU and T7 − MB on T cells using the prophylactic schedule (n ≥ 5 mice/group). **a** Cytotoxic T cell responses were induced by 5-FU and T7 − MB, as indicated by the lysis of EAC cells using the LDH method. **b** Increases in CD8^+^ and CD4^+^ T cells in splenic lymphocytes were induced by 5-FU and T7 − MB, as determined by flow cytometry. **c** Flow cytometry results of CD8^+^ T cells in splenic lymphocytes in the PBS control (left) and 5-FU and T7 − MB combination (right) groups. **d** Flow cytometry results of CD4^+^ T cells in splenic lymphocytes in the PBS control (left) and 5-FU and T7 − MB combination (right) groups. The data represent the mean ± SE; *P < 0.05, **P < 0.01 and ***P < 0.001 compared to the PBS control group

**Fig. 7:**
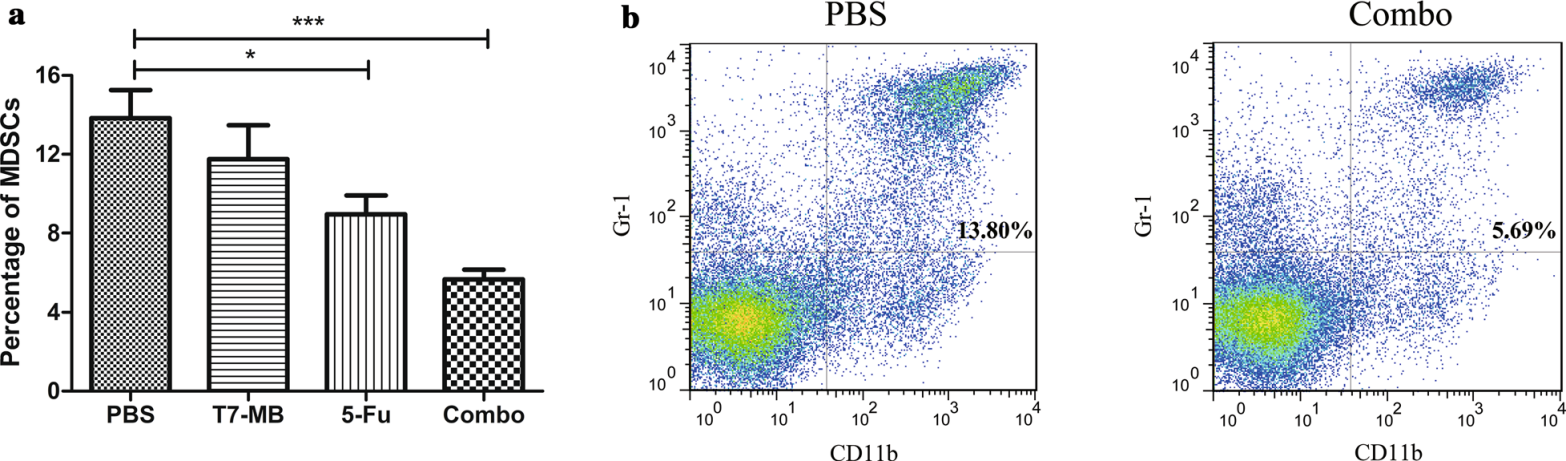
Additive effects of 5-FU and T7 − MB on MDSCs using the prophylactic schedule (n ≥ 5 mice/group). **a** Decreases in MDSCs in splenic lymphocytes were induced by 5-FU and T7 − MB, as determined by flow cytometry. **b** Flow cytometry results of MDSCs in splenic lymphocytes in the PBS control (left) and 5-FU and T7 − MB combination (right) groups. The data represent the mean ± SE; *P < 0.05 and ***P < 0.001 compared to the PBS control group

The CTL activities of the BALB/c mice were first investigated to uncover the mechanisms exerted by the simultaneous application of 5-FU and T7 − MB administered on the prophylactic schedule, and the results are illustrated in Fig. 6a. Although 5-FU alone induced CTLs to a certain degree, the difference between 5-FU and the PBS control was not significant. As expected, CTLs activated by the combination treatment group had obviously higher cytotoxicity values (48.86%) than those of CTLs activated by 5-FU (21.37%) and T7 − MB (38.99%) alone. Variances in the T cell subsets of mice splenic lymphocytes were then demonstrated by flow cytometry. As shown in Fig. 6b, c, the percentage of CD3^+^/CD8^+^ T cells in the T7 − MB group (15.25%) was higher than those in the 5-FU (13.02%) and PBS (10.18%) groups. The CD8^+^ T cell ratio increased significantly to 18.78% in the combination treatment group compared to that in the 5-FU and T7 − MB groups, which was consistent with the CTL results. CD4^+^ T cells had an outcome similar to that of CD8^+^ T cells, as 24.06% of the lymphocytes referred to CD3^+^/CD4^+^ T cells in the combination treatment group, which was much more potent than that in the 5-FU (16.15%) and T7 − MB alone (18.59%) groups (Fig. 6b, d). In addition, the ratios of CD8^+^ and CD4^+^ T cells in untreated naïve BALB/c mice were 14.87 and 19.01%, respectively, which were approximately equal to those in the T7 − MB group (data not shown). We also examined the capacities of 5-FU and T7 − MB on the reductions of myeloid-derived suppressor cells (MDSCs). As displayed in Fig. 7, 5-FU alone had stronger inhibitory effects on MDSCs than T7 − MB alone, while the combination treatment further decreased the MDSCs ratio to 5.67%. Untreated naïve BALB/c mice had merely 5.92% MDSCs in their splenocytes (data not shown), which was similar to that in the combination treatment group.

### Synergistic effects of 5-FU and T7 − MB on tumor inhibition using the therapeutic schedule

**Fig. 8:**
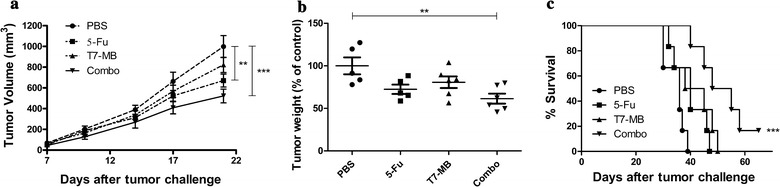
Additive effects of 5-FU and T7 − MB on tumor inhibition using the therapeutic schedule (n ≥ 5 mice/group). **a** Tumor growth curves displaying tumor volumes, where the dimensions were measured twice a week until day 21 (two-way ANOVA). **b** Tumor weights were determined at the time of sacrifice on day 21, where the PBS control (100%) was 0.94 ± 0.09 g. **c** Survival curves indicating that post-treatment effects on the tumor-bearing mice lasted more than 70 days (log-rank test). The data represent the mean ± SE; **P < 0.01 and ***P < 0.001 compared to the PBS control group

**Fig. 9:**
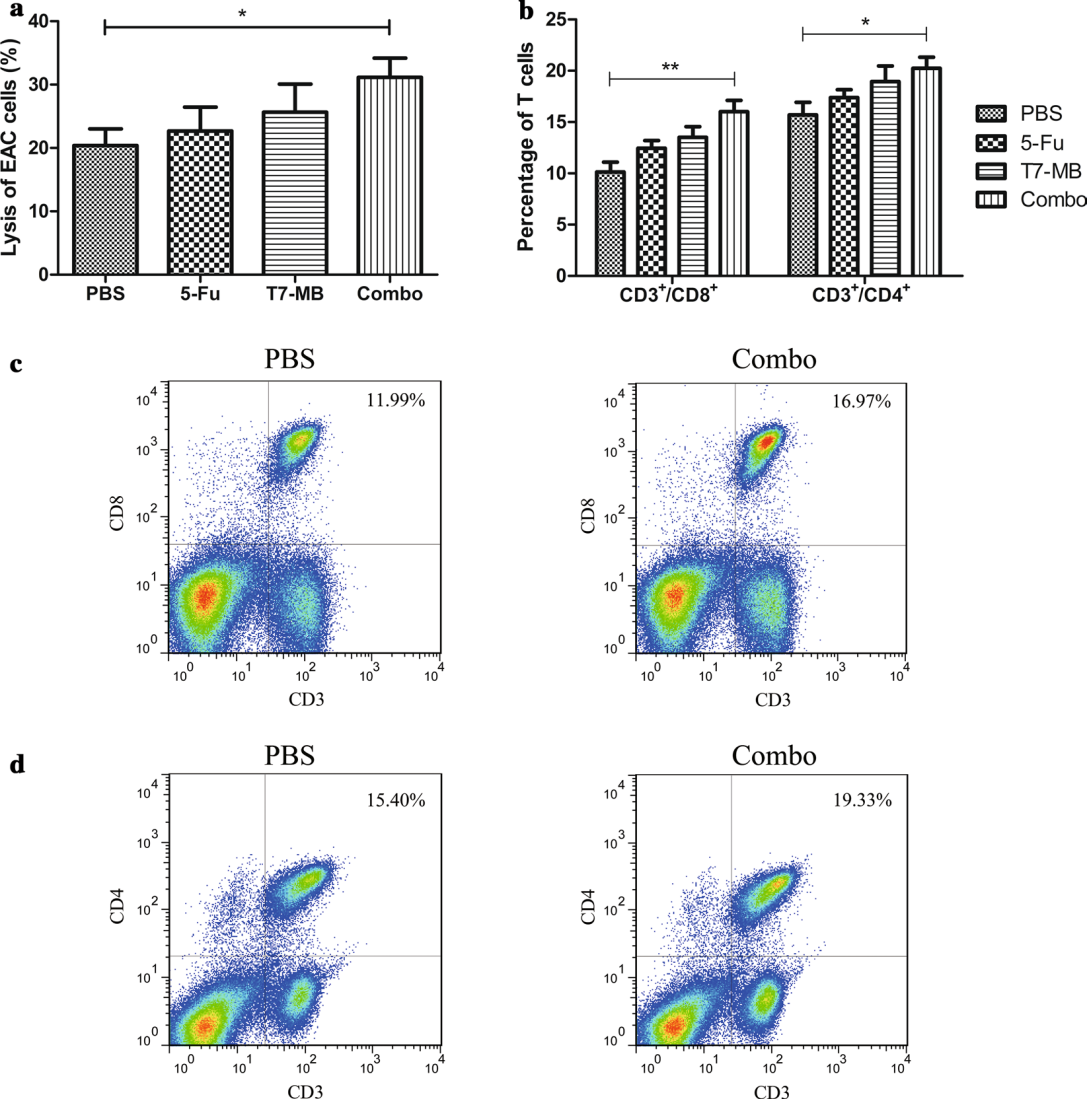
Additive effects of 5-FU and T7 − MB on T cells using the therapeutic schedule (n ≥ 5 mice/group). **a** Cytotoxic T cell responses were induced by 5-FU and T7 − MB, as indicated by the lysis of EAC cells using the LDH method. **b** Increases in CD8^+^ and CD4^+^ T cells in splenic lymphocytes were induced by 5-FU and T7 − MB, as determined by flow cytometry. **c** Flow cytometry results of CD8^+^ T cells in splenic lymphocytes in the PBS control (left) and 5-FU and T7 − MB combination (right) groups. **d** Flow cytometry results of CD4^+^ T cells in splenic lymphocytes in the PBS control (left) and 5-FU and T7 − MB combination (right) groups. The data represent the mean ± SE; *P < 0.05 and **P < 0.01 compared to the PBS control group

The therapeutic effects of the vaccines were assessed by subcutaneously challenging BALB/c mice with tumor cells, followed by the drug administration of T7 − MB. Figure 8a shows that 5-FU and T7 − MB administered on the therapeutic schedule exerted some inhibitory effects on tumor size and volume. The simultaneous administration of 5-FU and T7 − MB gradually induced significant tumor shrinkage from days 1 through day 21 compared with that induced by 5-FU or T7 − MB alone. In total, the tumor weights in the combination treatment group were 61.47% lower than those in the PBS control group, which was much lower than those in the 5-FU (72.45%) and T7 − MB (80.72%) groups (Fig. 8b). For the long-term survival rates, the final deaths of mice in the control, 5-FU and T7 − MB groups occurred on days 39, 47 and 50, respectively. Meanwhile, 16.67% of mice in the combination treatment group survived longer than 70 days (Fig. 8c). As shown in Fig. 9a, CTLs activated by the combination treatment displayed remarkably higher cytotoxicity (31.15%) than those in the PBS control group (20.38%), while the CTLs of the 5-FU and T7 − MB alone groups were not significantly different from those of the PBS control group. Figure 9b–d shows T cell subsets in mouse splenic lymphocytes after drug administration, where the percentages of CD3^+^/CD8^+^ and CD3^+^/CD4^+^ T cells in the combination treatment group were obviously higher than those in the 5-FU and T7 − MB alone groups. However, T7 − MB had little impact on MDSCs, and no significant difference in MDSCs was detected between the combination treatment group and the 5-FU alone group (data not shown).

## Discussion

Gastric cancer is the fifth leading cause of cancer and the third leading cause of death from cancer, comprising 7% of cases and 9% of deaths [14]. Traditional treatment for gastric cancer may include surgery, chemotherapy and radiation therapy. Currently, new approaches, especially immunotherapy, are being intensively investigated due to their advantages of effective targeting and few side effects [15]. In particular, trastuzumab represents the first target molecule that improves overall survival in human epithelial growth factor receptor-2 (HER2)-positive gastric cancer patients [16, 17]. A range of tyrosine-kinase inhibitors (sunitinib, apatinib, lapatinib, etc.) and monoclonal antibodies (bevacizumab, pertuzumab, etc.), aimed at vascular epithelial growth factor receptor (VEGFR), epithelial growth factor receptor (EGFR), the PI3K-AKT-mTOR pathway and the HGF-c-Met pathway, are also under investigation with promising results [18–20]. Immune checkpoint inhibitors, which represent a landmark innovation in malignant melanoma and non-small cell lung cancer therapies, are another measure to effectively treat gastric cancer [21]. A phase I clinical trial has been completed on a population of patients with PD-L1-positive gastric cancer, and pembrolizumab (a PD-1 antibody) has antitumor activity and manageable toxicity, warranting further phase II and III trials [22].

Tumor vaccines also play an important role in cancer therapy by stimulating the immune system to find and kill tumor cells. Sipuleucel-T, the first cell-based immunotherapeutic vaccine approved by the FDA, has been shown to improve the survival of patients with asymptomatic or minimally symptomatic metastatic castration-resistant prostate cancer (mCRPC) via initiating immune responses to prostatic acid phosphate (PAP) [23]. Regarding gastric cancer, vaccines against *Helicobacter pylori* and VEGFR demonstrate favorable effects as both single immunotherapy agents and in combination with other chemotherapeutic agents [24, 25]. However, the development of gastric cancer vaccines is still preliminary because of the difficulties in identifying TAAs and adjuvants.

Our group has a strong background in researching tumor vaccines with applications of TLR7 agonists. We have demonstrated that conjugation of T7 and the OCT4 (octamer-binding transcription factor 4) protein was effective and safe in preventing tumor growth in xenografted mice [26]. We have also constructed gastric cancer vaccines with T7 and the MG7-Ag tri-epitope, displaying some effects on generating CTLs and ADCC-mediating antibodies recognizing MG7-Ag [8]. Herein, to improve the efficiency of our vaccines, we developed novel gastric cancer vaccines utilizing MAPs of the MG7-Ag epitope (with either a linear or branched architecture). Branched MAPs aimed at enhancing immune responses against tumor cells, including MAPs based on CTL epitopes of human telomerase reverse transcriptase (hTERT), are clearly observable [27]. In vitro, our small-molecule compound T7 improved innate immunity in BMDCs in a concentration-dependent manner by inducing the inflammatory mediators TNF-α and IL-12. The combination of ML or MB with T7 yielded much more potent effects, although neither ML nor MB alone impacted cytokine secretion to any notable extent. Moreover, the branched architecture of the MG7-Ag epitope and its chemical conjugation to T7 (T7 − MB) had the most notable BMDC stimulatory activity (Fig. 3). Cytokines can further activate the expression of costimulatory molecules to assist interactions between antigen-presenting cells (APCs) and T and B cells [28]. Thus, we further investigated the impacts of our vaccines on adaptive immunity in vivo, assessing CTLs, antibodies, etc. Figure 4b–d suggests that T7 − MB elicited remarkable T lymphocyte cytotoxicity and IgG antibodies to specifically lyse EAC cancer cells. Indeed, T7 − MB was the most effective agent at reducing the tumor burden in vivo (Fig. 4a), which was also consistent with the in vitro results. We have reported the importance of covalently attaching the TLR7 agonist and the MG7-Ag epitope to designing gastric cancer vaccines [8]. Here, we proved that the branched MAP structure could further elevate the efficacies of tumor inhibition and immune stimulation, leading to a brand-new vaccine, T7 − MB.

Recently, immunochemotherapy, achieved by combining a chemotherapeutic agent with an immune-modulating agent, has become an appealing approach to cancer therapy [29]. Heterologous strategies, i.e., combination of remedies that activate immunity via different mechanisms, are distinctly preferred over homologous strategies because they not only require decreased drug dosages and reduce side effects but they also reduce chemo- and immunoresistance incidence rates [30]. For example, Iinuma et al. conducted a phase I clinical study on esophageal cancer patients, certifying the safe and satisfactory long-term therapeutic outcomes of peptide vaccination plus chemoradiation [31]. Although chemotherapies are commonly considered to act via cytotoxic pathways, accumulating evidence proves that immune responses can also be elicited by chemotherapeutic agents, contributing to the final antitumor effects [32, 33]. 5-Fluorouracil (5-FU), a clinically well-established medication, is administered either systemically for the treatment of colorectal, stomach and breast cancers or topically for skin cancer treatment. 5-FU is also reported to potently and selectively kill tumor-associated MDSCs and improve T cell-dependent responses in vivo [34]. MDSCs promote tumor growth by favoring tumor cell survival and creating an immunosuppressive microenvironment, which may severely destroy the efficacy of vaccines. We hypothesized that the inhibition of MDSCs by 5-FU was beneficial to the immunostimulatory properties generated by our vaccines. Therefore, we performed further research on the synergistic usage of our vaccine (T7 − MB) with 5-FU, attempting to fully understand their antitumor effects and mechanisms.

5-FU treatment (every 3 days after tumor implantation) demonstrated cytotoxicity by decreasing tumor sizes and weights and increasing survival time, although the effects were still weaker than those of T7 − MB vaccination. Substantially more significant results were observed when 5-FU and T7 − MB were applied in combination (Fig. 5). Furthermore, the CTL activity of the combination group was clearly higher than that in both the 5-FU and T7 − MB groups (Fig. 6a). However, no differences were observed between the groups with and without 5-FU in the determination of MG7-Ag antibody titers and ADCC activity (data not shown), indicating that 5-FU improved the T cell responses induced by T7 − MB rather than improving B cell responses. The CD8^+^ and CD4^+^ subsets are the two main T cells populations and play critical roles in host defenses against cancer. Upon stimulation, naïve CD8^+^ T cells can differentiate into memory and effector CD8^+^ T cells, while naïve CD4^+^ T cells can differentiate into T helper (Th) type 1, Th2 and Th17 cells [35]. As displayed in Fig. 6b–d, compared to the PBS control, T7 − MB vaccination markedly augmented the proportions of both CD3^+^/CD8^+^ and CD3^+^/CD4^+^ T cells in splenocytes, which were further elevated by the combinational usage of 5-FU despite the limited function of 5-FU alone. Regarding MDSCs, 5-FU depressed the ratio of CD11b^+^/Gr-1^+^ MDSCs in splenocytes treated with T7 − MB (Fig. 7), which contributed to the synergistic antitumor potency. In addition to the prophylactic benefits, therapeutic tumor vaccines are also prospective. For example, GTL001 induced strong antigen-specific T cell responses and tumor eradications in a curative strategy when adjuvanted with a TLR7 agonist [36]. Similarly, we detected the therapeutic consequences of our vaccine, T7 − MB, as well as those of 5-FU. Even though T7 − MB and 5-FU alone did not sufficiently impact tumor repression, tumors in the combination group were significantly different from those in the PBS control group (Fig. 8). The CTL activity and percentages of CD3^+^/CD8^+^ and CD3^+^/CD4^+^ T cells were also ameliorated when mice were treated with T7 − MB and 5-FU together (Fig. 9).

## Conclusions

In conclusion, we certified the application of TLR7 agonists and the MAP structure in the design of gastric cancer vaccines, with T7 − MB providing benefits to tumor-bearing mice via CTLs and ADCC-mediating antibodies recognizing MG7-Ag. In addition, the synergistic antitumor functions of T7 − MB and 5-FU were carefully examined, revealing that the prophylactic schedule was much more efficacious than the therapeutic schedule, and T cell responses played more important roles than B cell responses. MDSCs down-regulated by 5-FU were also important for promoting the effects of T7 − MB. Although the specific dosage regimen of T7 − MB could be further optimized to maximize antitumor outcomes, we herein demonstrated the potentials of the construction and immunochemotherapy of our novel vaccine in cancer treatment schemes.

## Additional files

**Additional file 1.** TIF High performance liquid chromatography (a) and mass spectrometry (b) of ML.

**Additional file 2.** TIF High performance liquid chromatography (a) and mass spectrometry (b) of T7 − ML.

**Additional file 3.** TIF High performance liquid chromatography (a) and mass spectrometry (b) of MB.

**Additional file 4.** TIF High performance liquid chromatography (a) and mass spectrometry (b) of T7 − MB.

**Additional file 5.** TIF The presence of MG7-Ag in EAC cells confirmed by western blot. 4T1 mouse breast cancer cell line was used as a negative control.

## Abbreviations

TLR: Toll-like receptor
MDSC: Myeloid-derived suppressor cell
MG7-Ag: Monoclonal gastric cancer 7 antigen
5-FU: 5-Fluorouracil
CTL: Cytotoxic T lymphocyte
ADCC: Antibody-dependent cell-mediated cytotoxicity
LDH: Lactate dehydrogenase
BMDC: Bone marrow dendritic cell
EAC: Ehrlich ascites carcinoma
TAA: Tumor-associated antigen
MAP: Multiple antigen peptide
