# Supplementary figures and images for: Gastric cancer vaccines synthesized using a TLR7 agonist and their synergistic antitumor effects with 5-fluorouracil
Source: J Transl Med. 2018 May 8;16:120. doi: 10.1186/s12967-018-1501-z (PMC5941430; doi:10.1186/s12967-018-1501-z)

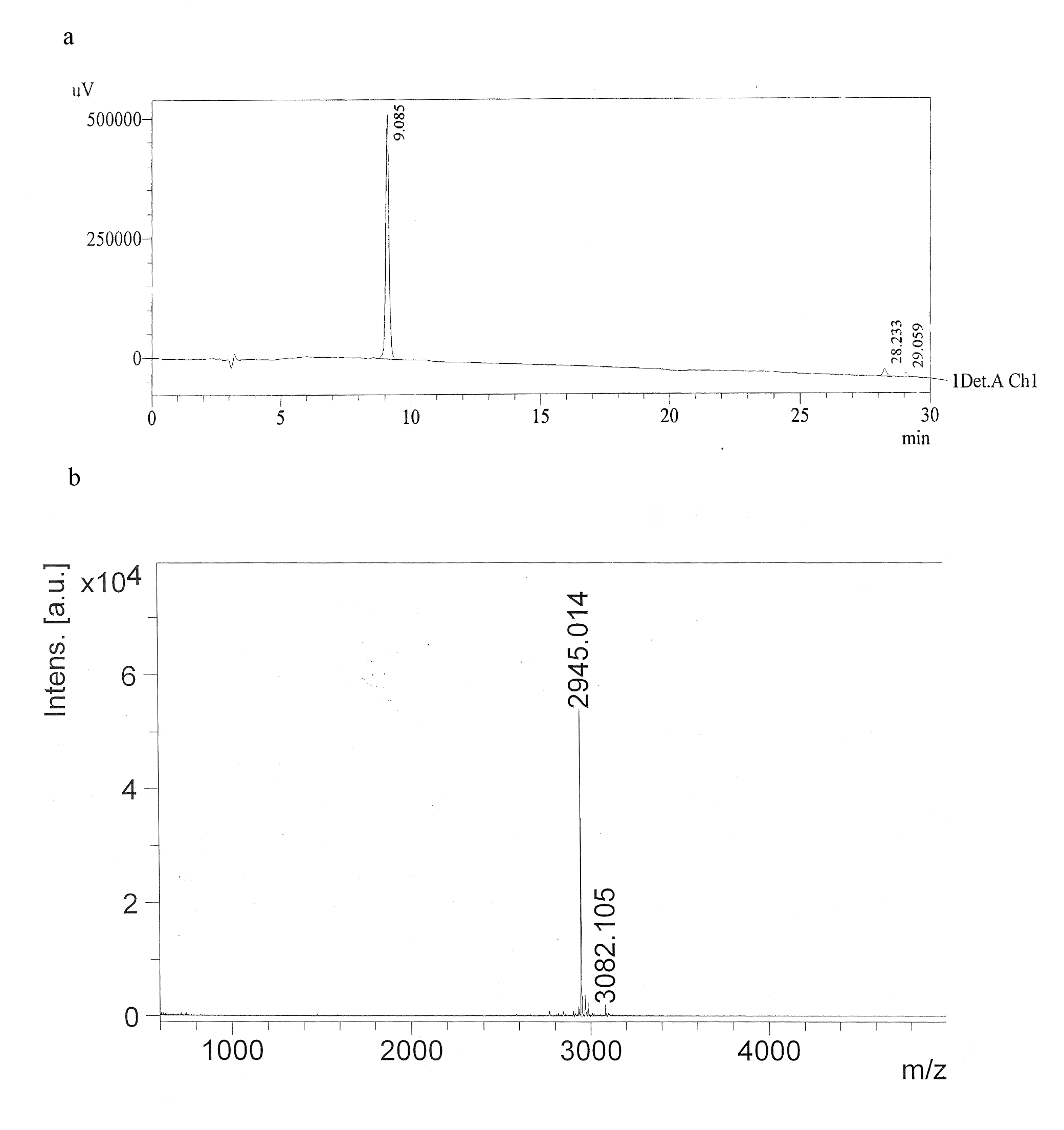

Supplement: Supplementary file 1 — Additional file 1. TIF High performance liquid chromatography (a) and mass spectrometry (b) of ML. [file 12967_2018_1501_MOESM1_ESM.tif]

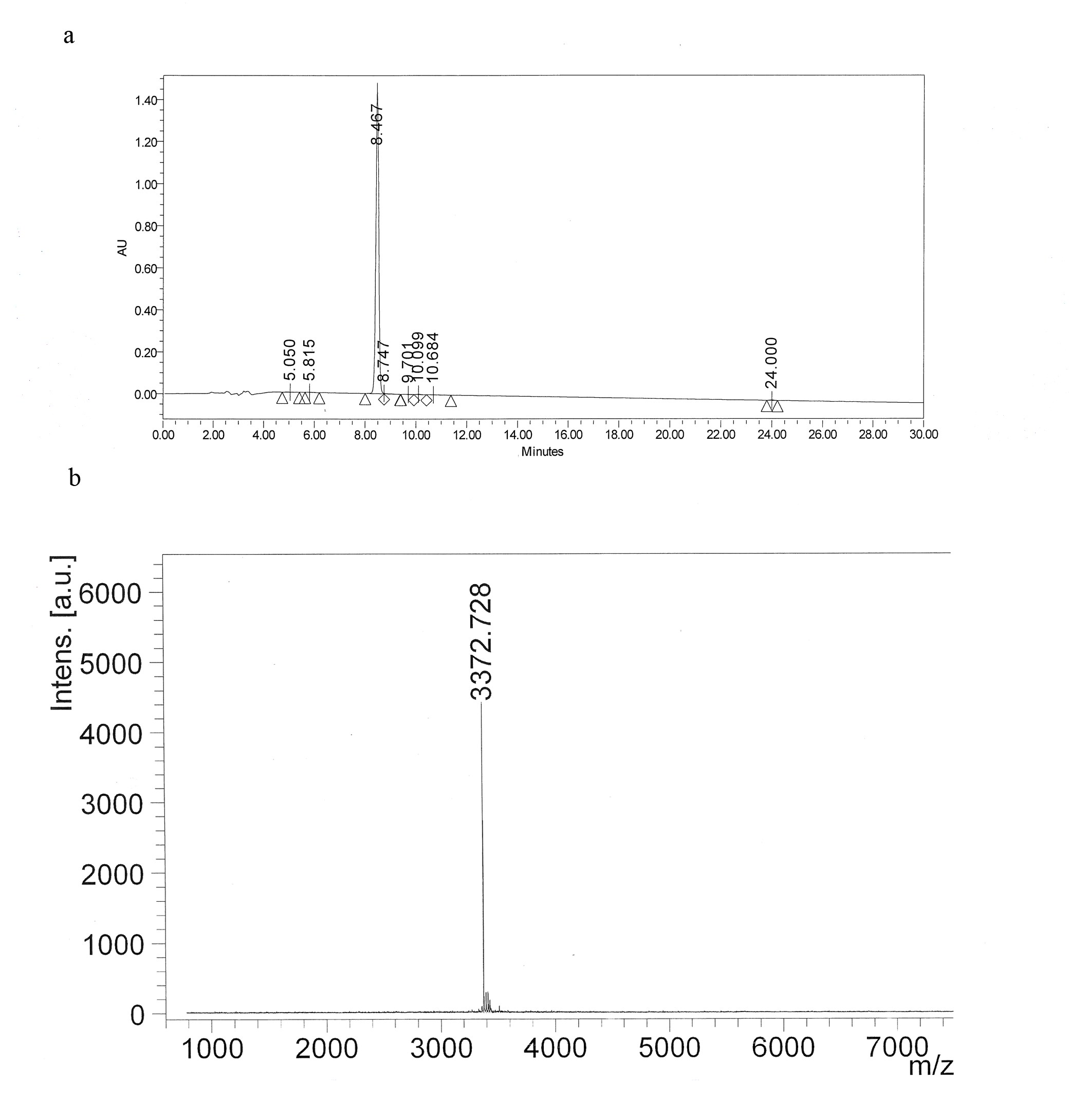

Supplement: Supplementary file 2 — Additional file 2. TIF High performance liquid chromatography (a) and mass spectrometry (b) of T7 − ML. [file 12967_2018_1501_MOESM2_ESM.tif]

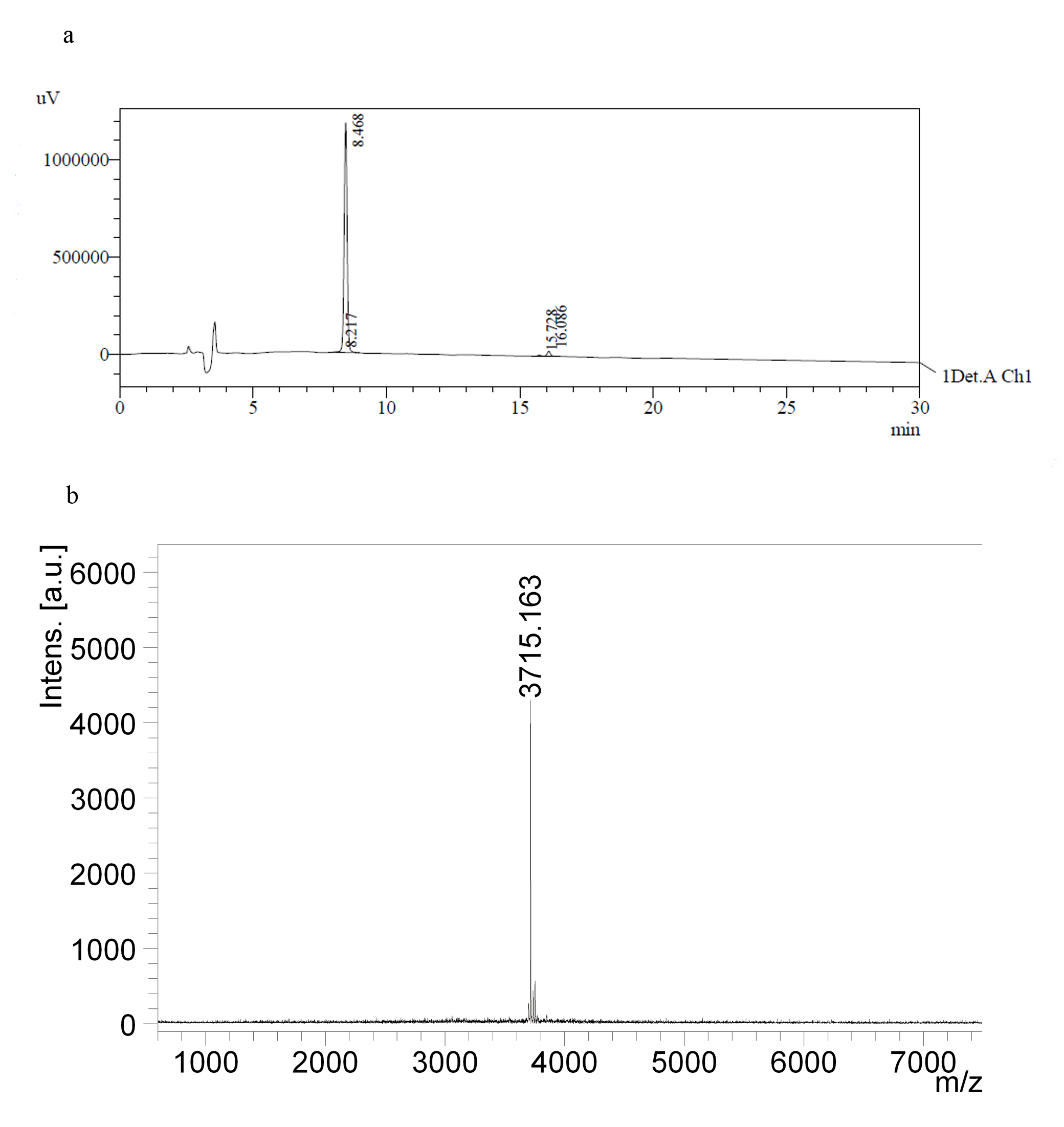

Supplement: Supplementary file 3 — Additional file 3. TIF High performance liquid chromatography (a) and mass spectrometry (b) of MB. [file 12967_2018_1501_MOESM3_ESM.tif]

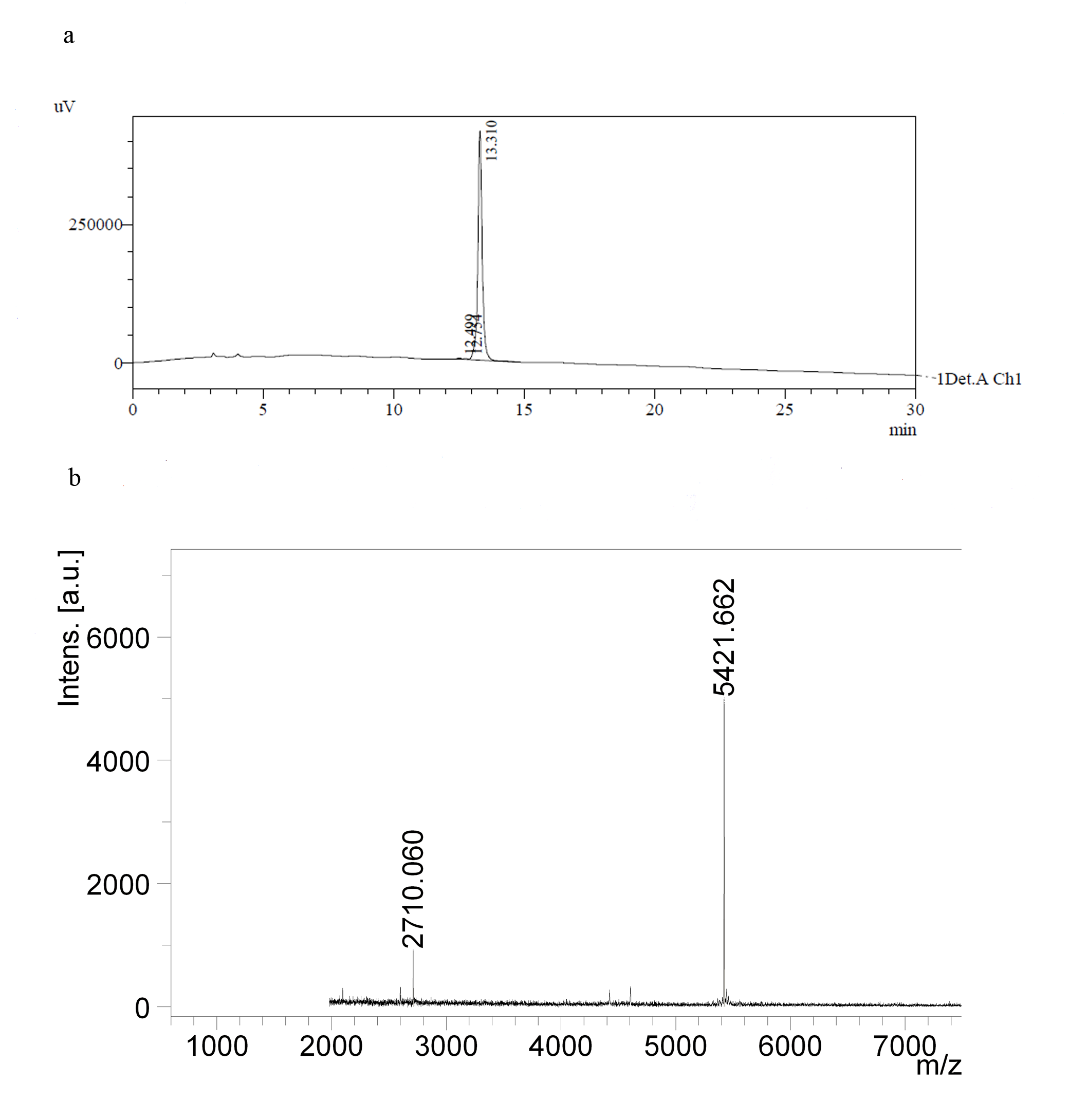

Supplement: Supplementary file 4 — Additional file 4. TIF High performance liquid chromatography (a) and mass spectrometry (b) of T7 − MB. [file 12967_2018_1501_MOESM4_ESM.tif]

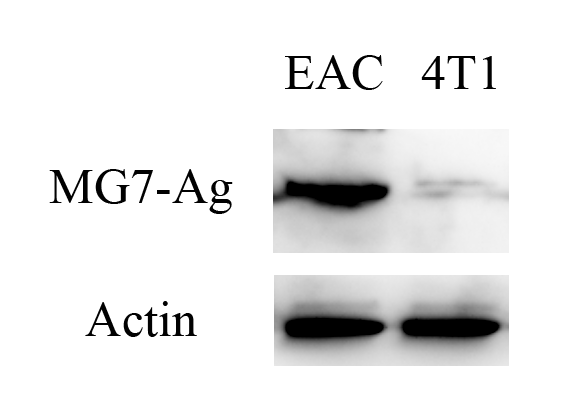

Supplement: Supplementary file 5 — Additional file 5. TIF The presence of MG7-Ag in EAC cells confirmed by western blot. 4T1 mouse breast cancer cell line was used as a negative control. [file 12967_2018_1501_MOESM5_ESM.tif]
